# Neural Network Prediction of ICU Length of Stay Following Cardiac Surgery Based on Pre-Incision Variables

**DOI:** 10.1371/journal.pone.0145395

**Published:** 2015-12-28

**Authors:** Rocco J. LaFaro, Suryanarayana Pothula, Keshar Paul Kubal, Mario Emil Inchiosa, Venu M. Pothula, Stanley C. Yuan, David A. Maerz, Lucresia Montes, Stephen M. Oleszkiewicz, Albert Yusupov, Richard Perline, Mario Anthony Inchiosa

**Affiliations:** 1 Department of Surgery, New York Medical College, Valhalla, New York, United States of America; 2 Department of Anesthesiology, New York Medical College, Valhalla, New York, United States of America; 3 Department of Pharmacology, New York Medical College, Valhalla, New York, United States of America; 4 Revolution Analytics, Inc., Mountain View, California, United States of America; 5 The SAS Institute, Cary, North Carolina, United States of America; Jiangnan University, CHINA

## Abstract

**Background:**

Advanced predictive analytical techniques are being increasingly applied to clinical risk assessment. This study compared a neural network model to several other models in predicting the length of stay (LOS) in the cardiac surgical intensive care unit (ICU) based on pre-incision patient characteristics.

**Methods:**

Thirty six variables collected from 185 cardiac surgical patients were analyzed for contribution to ICU LOS. The Automatic Linear Modeling (ALM) module of IBM-SPSS software identified 8 factors with statistically significant associations with ICU LOS; these factors were also analyzed with the Artificial Neural Network (ANN) module of the same software. The weighted contributions of each factor (“trained” data) were then applied to data for a “new” patient to predict ICU LOS for that individual.

**Results:**

Factors identified in the ALM model were: use of an intra-aortic balloon pump; O_2_ delivery index; age; use of positive cardiac inotropic agents; hematocrit; serum creatinine ≥ 1.3 mg/deciliter; gender; arterial pCO_2_. The r^2^ value for ALM prediction of ICU LOS in the initial (training) model was 0.356, p <0.0001. Cross validation in prediction of a “new” patient yielded r^2^ = 0.200, p <0.0001. The same 8 factors analyzed with ANN yielded a training prediction r^2^ of 0.535 (p <0.0001) and a cross validation prediction r^2^ of 0.410, p <0.0001. Two additional predictive algorithms were studied, but they had lower prediction accuracies. Our validated neural network model identified the upper quartile of ICU LOS with an odds ratio of 9.8(p <0.0001).

**Conclusions:**

ANN demonstrated a 2-fold greater accuracy than ALM in prediction of observed ICU LOS. This greater accuracy would be presumed to result from the capacity of ANN to capture nonlinear effects and higher order interactions. Predictive modeling may be of value in early anticipation of risks of post-operative morbidity and utilization of ICU facilities.

## Introduction

The availability of advanced statistical software for predictive analytical investigations has led to many applications in clinical outcomes research. Artificial neural networks have been used in attempts to weight the contribution of various factors, and their interactions, to a particular medical diagnosis, classification, or clinical outcome. The networks use algorithms that are derived from preexisting data to arrive at the smallest prediction errors when applied to the same combination of factors in newly acquired data. Amato et al. [1] have reviewed the rather extensive literature that has focused on neural network applications in medical diagnosis. They also provide an excellent introduction to the mathematical foundation and design of neural networks, and how they are suggested to simulate the “learning” and “generalization” properties of human neural networks. For example, such networks have been applied to diagnosis of coronary artery syndromes [2,3], interpretation of electrocardiograms [4,5] classification of hemodynamic states in pregnancy [6], prediction of intensive care unit length of stay (ICU LOS) in trauma patients[7], and prediction of an adverse outcome in cardiac surgery [8].

Tu and Guerriere [9] appear to be among the first to apply a neural network model to predict ICU LOS following cardiac surgery. They evaluated 15 pre-operative factors in relation to a binary measure, i.e., greater or less than 48 hours of ICU care. The area under the receiver operator curve (AUC) for their training set was 0.7094, S.E. 0.0224. They tested the trained network on data from an independent set of patients; the AUC for the test set was 0.6960, S.E. 0.0227.

Buchman et al. [10] also evaluated prediction accuracy for a binary outcome measure of ICU LOS. They compared several neural network models with a multiple logistic regression model. Of patients in a general surgical ICU that were surviving on the third postoperative day, they evaluated the prediction of those that would be discharged between days 3 through 6 and those that would require more than 7 days of ICU care. Data for the 11 factors that were included in the prediction models were collected through ICU Day 3. The maximum sensitivity and specificity obtained with a neural network model in these predictions was 97% and 83%, respectively; r^2^ = 0.57. The multiple logistic regression model achieved a sensitivity and specificity of 72% and 62%, respectively; r^2^ = 0.26.

Rowan et al. [11] used artificial neural networks to study the influence of 32 demographic factors, medical and surgical histories, and clinical measures in relation to the need for an extended LOS in the post-cardiac surgery ICU. The factors encompassed the pre-operative (15 variables), intra-operative (2 variables), and post-operative (15 variables) periods. The immediate post-operative factors included 15 hemodynamic, respiratory and renal parameters, cognitive state, and blood chemistries and blood gas analyses. They evaluated a binary outcome of less than or greater than 24 hours LOS in the ICU. Ensembles of neural network models were found to strengthen predictive accuracies; the strongest model resulted in an AUC of 0.902 (sensitivity of 91%, specificity of 78%).

We have compared the predictive strength of a linear model to that of an artificial neural network in relation to LOS in the post-cardiac surgical ICU utilizing only pre-incision (i.e. prior to initiation of surgery) variables; the same variables were evaluated in both models. The studies noted above were designed to predict a binary outcome of a shorter or longer period of ICU stay. We have attempted to predict a continuous outcome in hours of ICU LOS. Also, some of the previous studies were not limited to pre-operative/pre-incision variables in their prediction models, but extended to factors derived from the intra-operative, post-operative and early ICU periods.

The primary objective of this study was to demonstrate the predictive strength that may be possible from pre-operative/pre-incision patient characteristics using a neural network model, and its superiority over a linear model; both models were generated with a commercially available statistical software package. It may be noted that statistical strengths could be identified even with a relatively modest patient sample size. It has also been our objective to qualify the perception that neural networks constitute “black boxes;” we have included details of the algorithms that allow each input variable to be tracked and interpreted for its positive, negative, synergistic or neutralizing contribution to the outcome variable.

We also carried out secondary comparative analyses of our data with two additional predictive analytical models, Decision Tree and Random Forest. These models have wide interest because of their inherent transparency in model development.

The odds ratio of our final neural network model to identify patients with the highest risk of prolonged ICU stays is also presented.

## Methods

This study was carried out with New York Medical College IRB approval and a Westchester Medical Center (Valhalla, New York) HIPAA waiver to collect medical record data, retrospectively. Data were collected with complete confidentiality and plan for de-identification from a total of 185 patients who underwent coronary artery bypass graft (CABG) or heart valve surgeries, or a combination of both. All patients underwent surgery with cardiopulmonary bypass. A total of 7 patients (3.8%) were excluded as follows: Expiration in the ICU, 1; unplanned return to the operating room from the ICU, 1; additional aortic surgery not including the valve, 3; one renal transplant patient that had remained intubated in the ICU for 7 days before cardiac surgery; one patient with data inadvertently collected twice. The “Automatic Linear Modeling” (ALM) module and the “Artificial Neural Network” (ANN) module of IBM SPSS 21 statistical software (New Orchard Road, Armonk, NY 10504) were used for the primary analyses. Secondary analyses used the “Decision Tree” module of IBM SPSS 22 software, and open source R software [12] was used for “Random Forest” analyses.

### Automatic Linear Modeling

**Fig 1:**
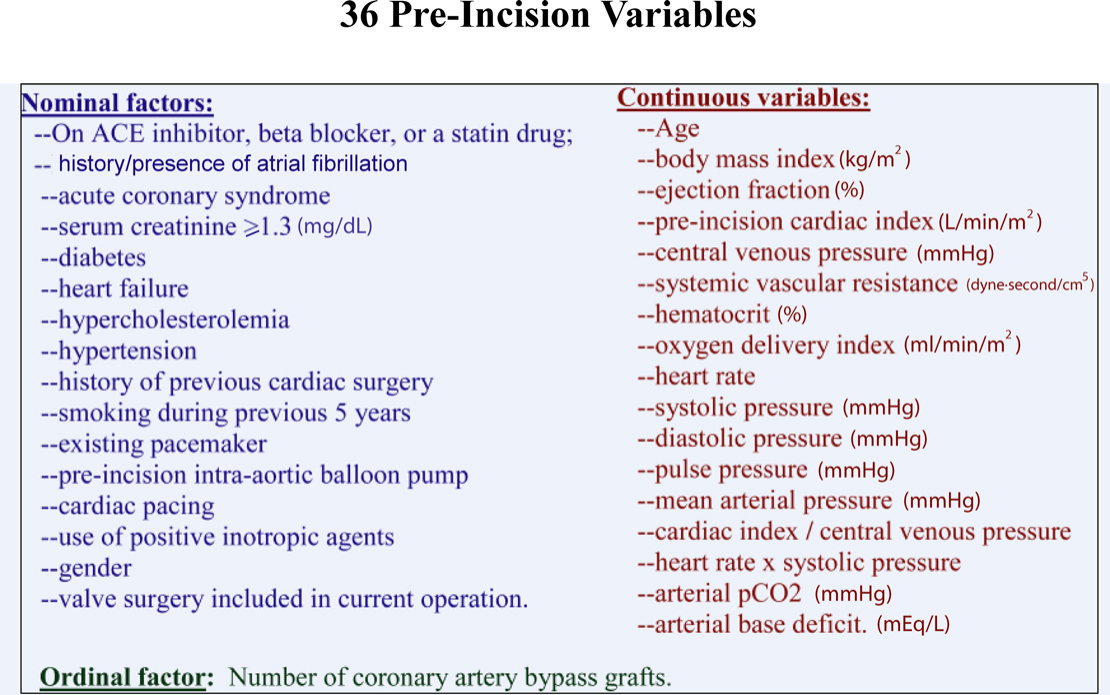
Pre-incision variables chosen for predictive analytical modeling from a data base of 185 cardiac surgical patients.

A set of 36 pre-incision/pre-operative demographic, anthropomorphic, hemodynamic, medical and cardiac surgical histories, and clinical interventions (Fig 1) were analyzed as input variables with the ALM module; hours of ICU LOS was the continuous output (target) variable. In our application, the model identified 8 pre-incision factors that were statistically associated with ICU LOS: Use of an intra-aortic balloon pump (IABP); O_2_ delivery index (ml/min/m^2^); age; use of positive cardiac inotropic agents; blood hematocrit level (%) (HCT); serum creatinine ≥ 1.3 mg/deciliter (dL); gender; arterial pCO_2_ (mm Hg). The software module preserves a file of the weighted contributions of each of the factors incorporated in the model. These weights represent a “trained” data set; these weights can then be applied to data for a new patient (“untrained data”) to predict ICU LOS for that individual.

The predictive accuracy of the trained weights on untrained data was evaluated by the “cross-validation” approach [13]. For this analysis, the data from 3 patients were excluded in training the entire remaining data; the weights were then applied to predict the ICU LOS for the 3 untrained patients. The data for those 3 patients were returned to the data set, and the process was repeated, excluding a new set of 3 patients each time. Thus, a total of approximately 60 analyses were carried out to get predicted ICU LOS for each patient.

### Neural Network Modeling

The same 8 variables that were identified by linear modeling to be statistically associated with ICU LOS were used for neural network modeling. Although it might have been possible to identify other variables that would give stronger predictive results for ICU LOS, we considered that a comparison with linear modeling would be best served by using the same variables in this demonstration study.

A considerable number of trials were run in our attempts to optimize the operator options in the design of a neural network with the IBM-SPSS software. Specific settings were as follows:

### Initial mode choice

Multilayer Perceptron

### Variable entry window

Nominal variables entered as, “Factors”

Ordinal variables entered as, “Factors”

Continuous variables entered as, “Covariates”

Outcome target variable entered as, “Dependent variable”

### Partitioning of the cases

All training of the model and cross validation were carried out with 90% of the cases assigned to training and 10% of the cases dedicated to testing (these are randomly assigned by the software with each run). The inclusion of test cases is essential to prevent “overtraining” of the model. The test cases are repeatedly used in the iterative process to optimize the predictive strength of the model.

### Rescaling of covariate

Standardized option: the mean is subtracted and the result is divided by the standard deviation; (*x*-mean)/*s*

### Architecture:

Minimum number of nodes in hidden layer, 1

Maximum number of nodes in hidden layer, 50

### Training criteria

Type of training: batch

Optimization algorithm: scaled conjugate gradient

Initial Lambda: 0.0000005

Initial Sigma: 0.00005

Interval center: 0

Interval offset: 0.5

### User missing values

Exclude

### Stopping rules

Maximum steps without a decrease in error: 5

Default options were used for any other choices.

As with the ALM module, the neural network module preserves a file of the weighted contributions of each of the factors incorporated in the model. These weights represent the trained data set, and can be applied to new untrained data. The cross-validation approach, as described above, was used with repeated sets of 3 patients to arrive at predicted values for ICU LOS for “untrained” data. There is a major difference between ALM and neural networks in regard to the generation of ICU LOS predictions in both the training output and the output in the cross-validation analyses. The linear modeling produces the identical prediction with repeated runs. In comparison, the neural network has an inherent instability (subtle variations) in the output predictions. The iterative analytical process is much more complex in the neural networks. A common approach to accommodate this instability is to use an ensemble of training sets to generate an average output prediction [14,15]. We used an ensemble of 10 trained sets in all predictions of ICU LOS. This procedure does not introduce any time restraints because of the speed of the computer processing of the algorithms. Our analyses were carried out with a common PC laptop computer with a 32-bit operating system, 2.10 GHz microprocessor and 3.00 GB of RAM. Individual training and cross-validation machine runs were typically completed in approximately 1.0 s with our size data sets.

## Results

### Automatic Linear Modeling

As noted above, 36 pre-incision variables (Fig 1) were analyzed with the ALM module. Eight variables were found to have a statistically significant (or close to statistically significant) correlation with ICU LOS. These included: Pre-incision use of an intra-aortic balloon pump; O_2_ delivery index; age; pre-incision use of positive cardiac inotropes; hematocrit; serum creatinine ≥ 1.3; gender; arterial pCO_2_. Their weighted coefficients to the final model, t statistic and p values for linear correlations, and fractional contributions to the final correlation with ICU LOS are presented in Table 1.

**Table 1:** ALM Fractional Contributions to the Final Correlation with ICU LOS.

Model Term	Coefficient (hrs)	t value	Significance(p)	Fractional Importance
**Intercept**	180.42	4.493	0.000	
**IABP = Yes**	0			
**IABP = No**	-37.94	3.304	0.001	0.243
**O**_**2**_ **Delivery Index**	-0.10	2.819	0.005	0.177
**Age**	0.76	2.676	0.008	0.160
**Inotropes = Yes**	0			
**Inotropes = No**	-55.64	2.485	0.014	0.138
**HCT**	-1.30	2.250	0.026	0.113
**Creatinine ≥ 1.3**	0			
**Creatinine < 1.3**	-12.97	1.715	0.088	0.066
**Male**	-10.29	1.556	0.122	0.054
**Female**	0			
**Arterial pCO**_**2**_	0.64	1.497	0.136	0.050

**Fig 2:**
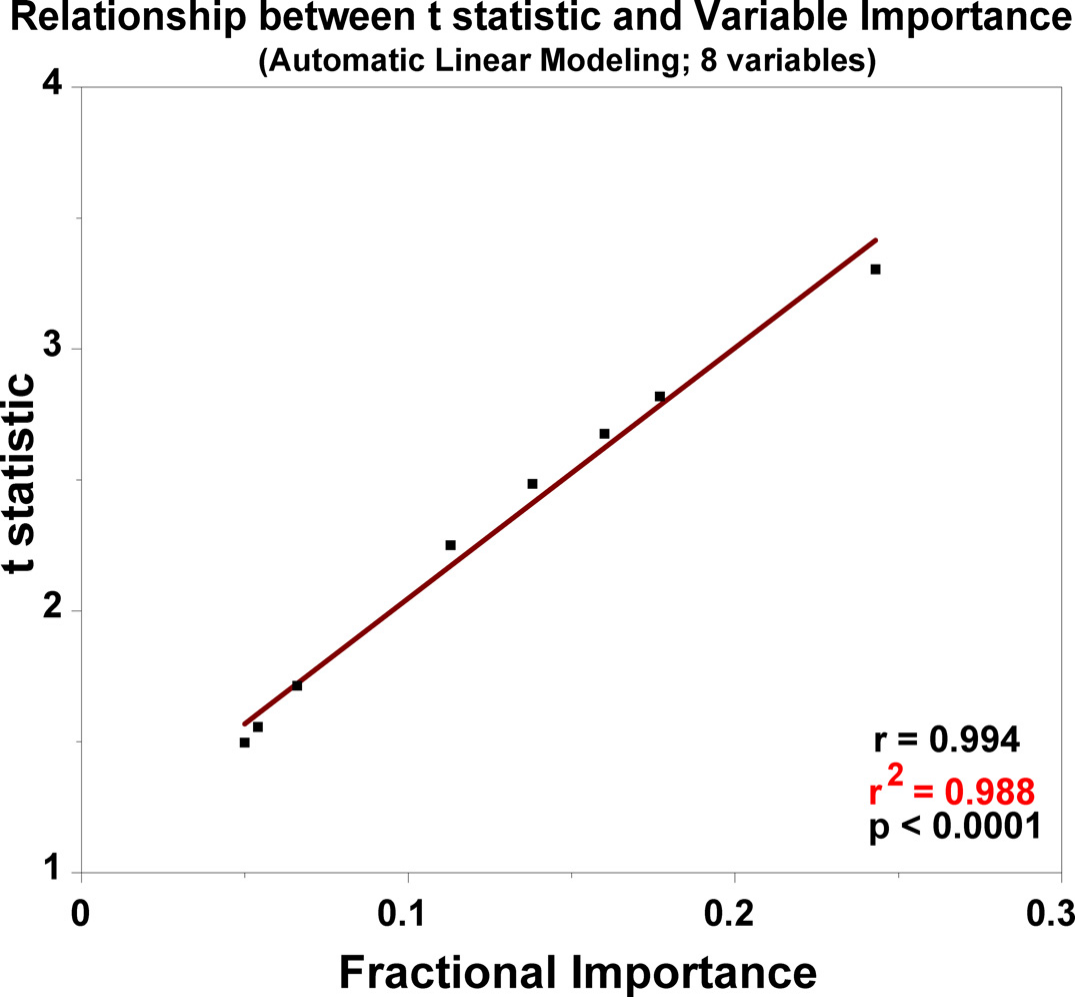
The correlation between the t statistic and the fractional importance of the 8 pre-incision factors that had the strongest associations with ICU length of stay.

The correlation between the t statistic and the fractional importance in the ALM model is shown in Fig 2. The application of the weighted coefficients to determination of ICU LOS is presented in S1 Appendix.

**Fig 3:**
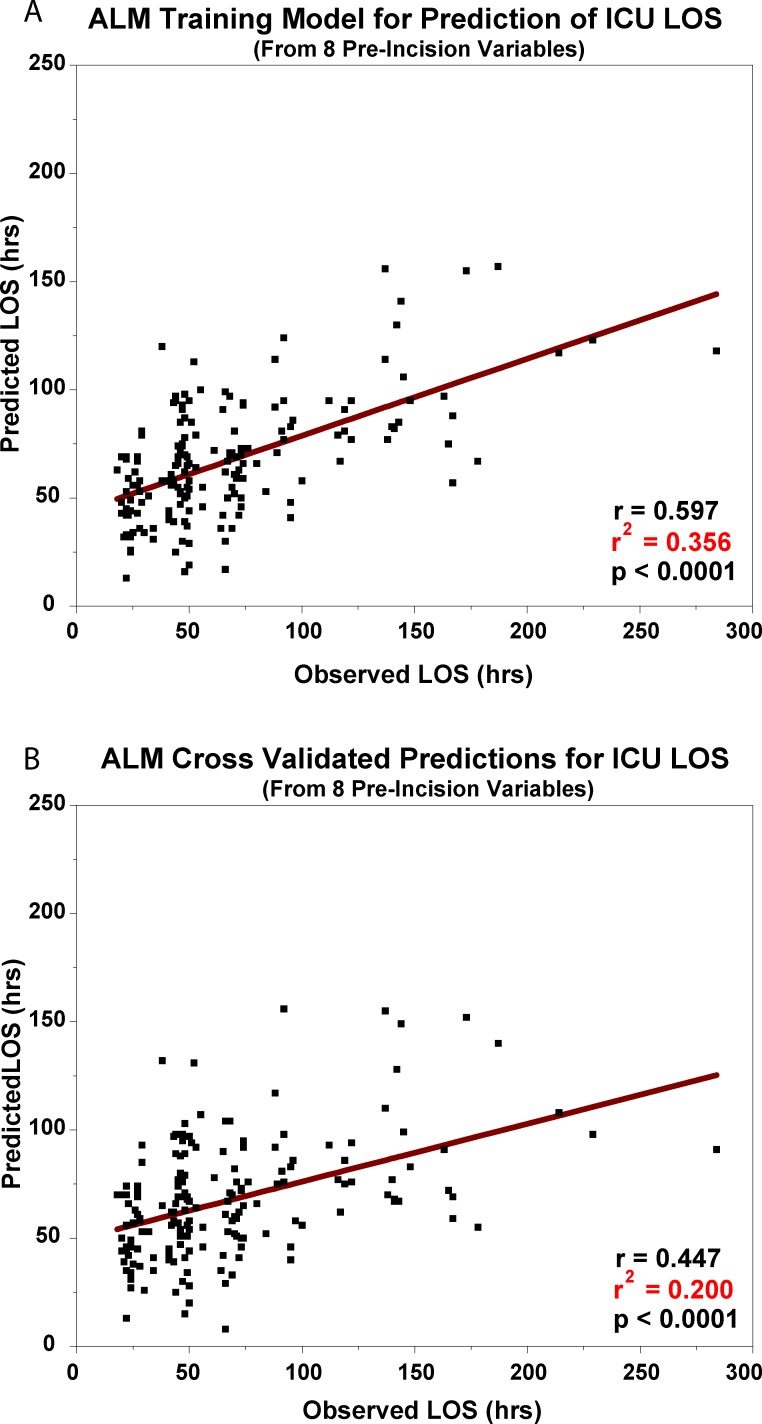
A. ALM Training Model for Prediction of ICU LOS. The regression relationship between the predicted ICU length of stay from 8 pre-incision factors and the observed ICU length of stay from the training model generated by the SPSS Automatic Linear Modeling module. B. ALM Cross-Validated Predictions for ICU LOS. The cross-validated result of the training model when applied to new ("untrained") patient data.

Of the 36 variables, only the 8 listed above were used to generate the correlation with LOS in the training set (Fig 3A), and their weighted contributions were preserved for the cross-validation of untrained data. The r^2^ value for the training set was 0.356, p <0.0001. The cross-validation results with untrained data are presented in Fig 3B. The r^2^ value for the final prediction was 0.200, p <0.0001.

### Neural Network Modeling

The same 8 variables identified by linear modeling were entered in the neural network module.

**Fig 4:**
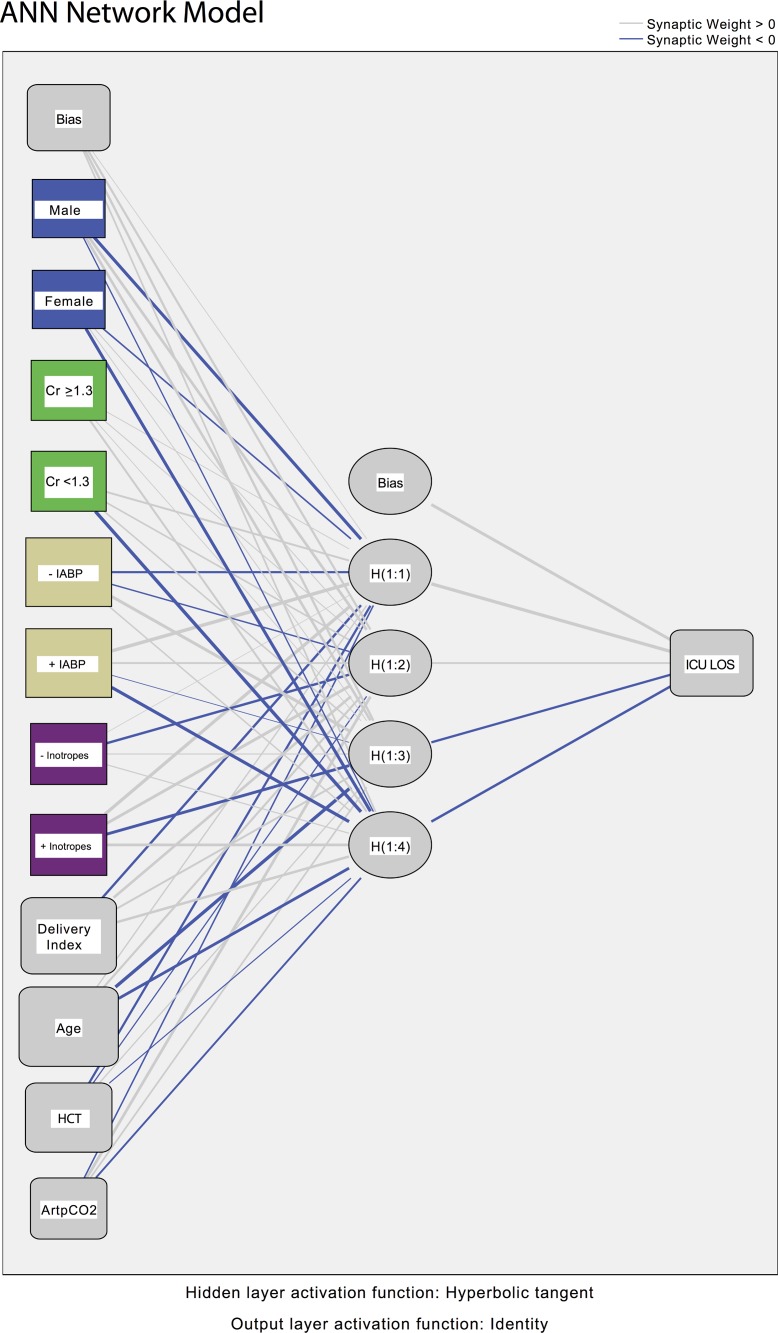
The architecture of the typical neural network utilized in the SPSS Artificial Neural Network module for the 8 pre-incision factors.

**Fig 5:**
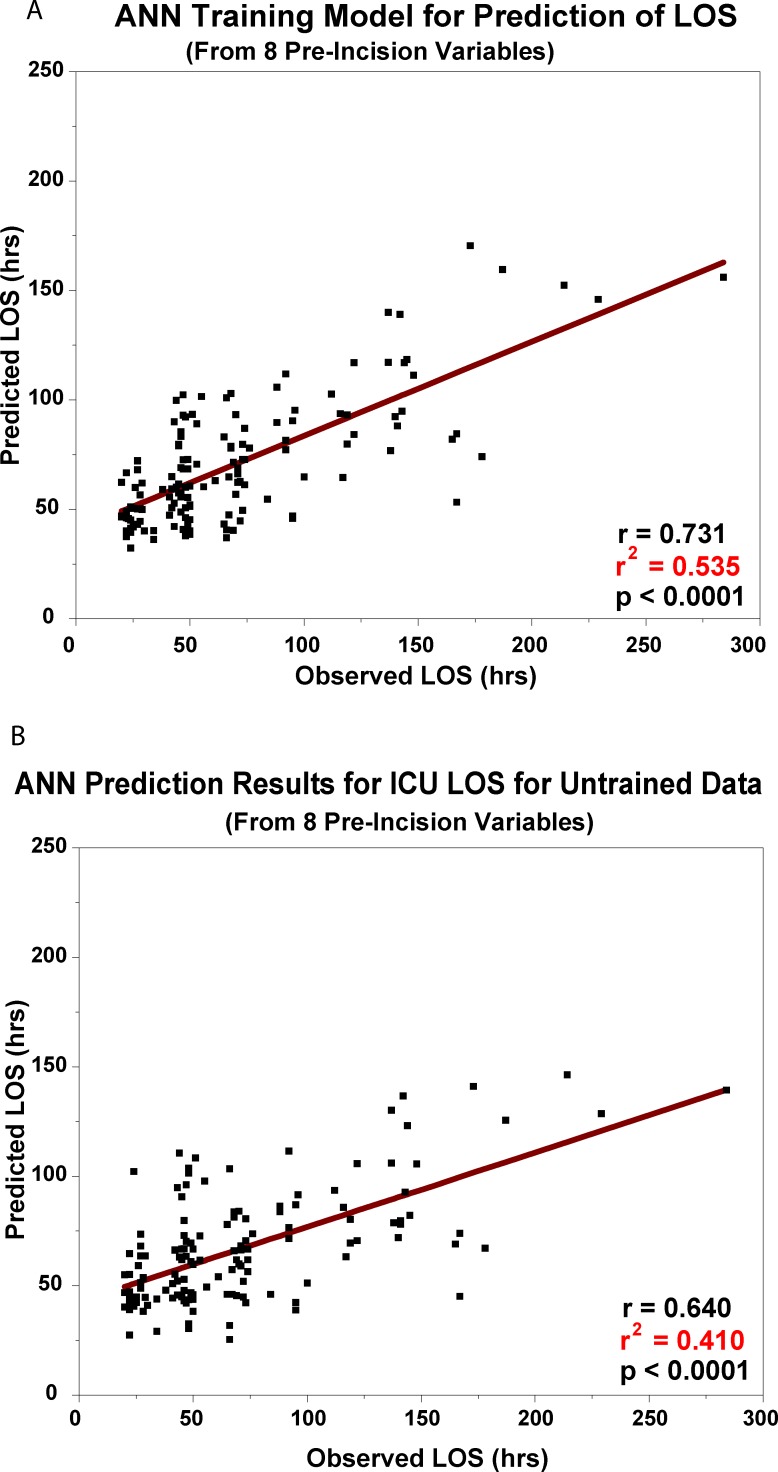
A. ANN Training Model for Prediction of ICU LOS. The regression relationship between the predicted ICU length of stay from 8 pre-incision factors and the observed ICU length of stay from the training model generated by the SPSS Artificial Neural Network module. B. ANN Prediction Results for ICU LOS for Untrained Data. The cross-validated result of the training model when applied to new ("untrained") patient data.

The typical model network generated by the software iterations is presented in Fig 4; 4 hidden nodes were typically employed by the software in the optimization process. The neural network predicted output for ICU LOS for the training set is presented in Fig 5A; the r^2^ for the correlation was 0.535, p <0.0001. As noted above, ensembles of trained data sets were used in cross-validation. The cross-validation results with untrained data with neural network analysis are presented in Fig 5B. The r^2^ value for the final prediction was 0.410, p <0.0001.

**Fig 6:**
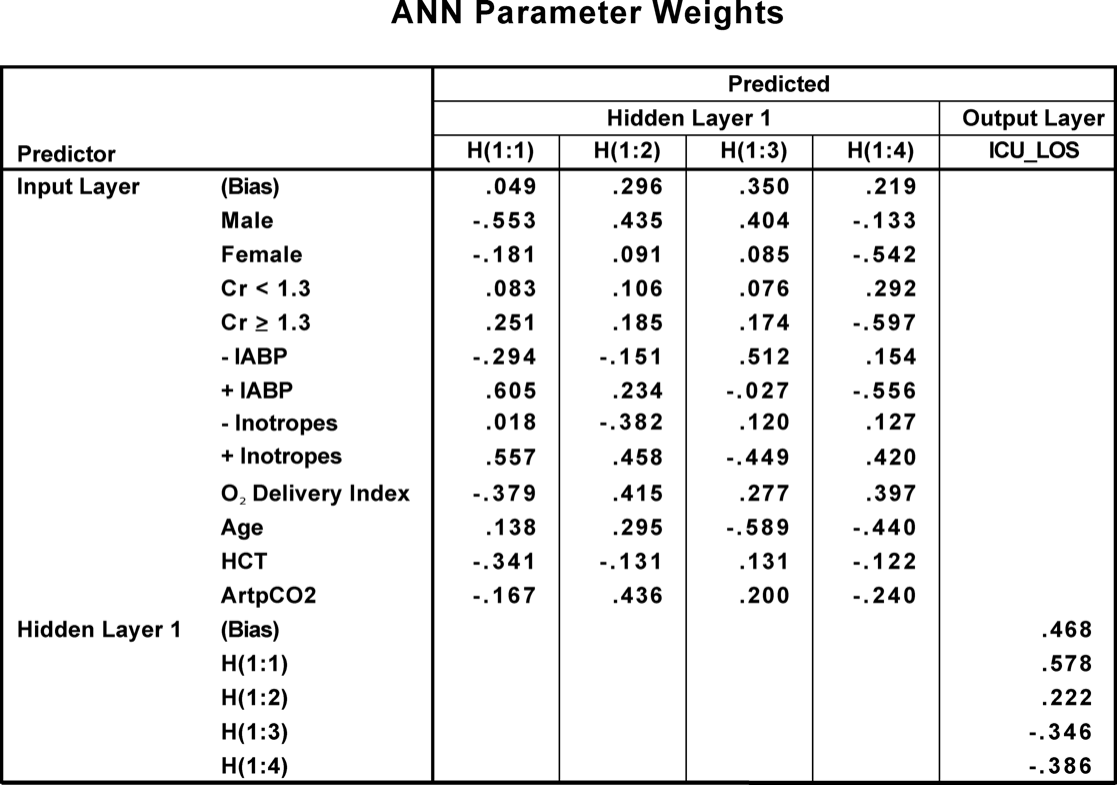
ANN Parameter Weights. The prediction weights generated by the neural network for each interaction among the 8 pre-incision factors ("input layer") and the 4 nodes ("hidden layer"), and the output weights of each node to the prediction of ICU length of stay; bias weights are also contributed from the input layer and the hidden layer.

A set of typical parameter weights preserved for the cross-validation analysis are presented in Fig 6. The formulas for the application of the parameter weights to the prediction of ICU LOS are presented in S2 Appendix. It should be noted that this represents the algorithm for the neural network.

**Fig 7:**
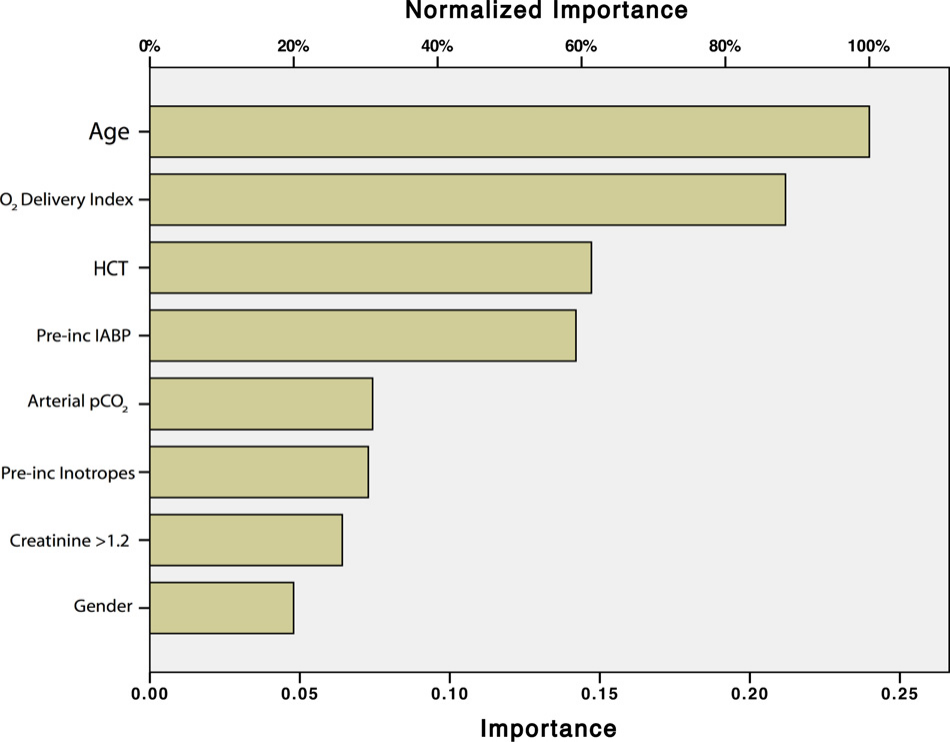
The relative importance of the 8 pre-incision factors to the anticipation of ICU length of stay (generated from the Artificial Neural Network algorithm).

The typical relative importance of the variables in the neural network modeling for this data set is presented in Fig 7; the algorithm for determination of predictor importance, also termed “sensitivity,” is presented in S3 Appendix.

We calculated the predicted odds ratio for a patient falling in the upper quartile of the observed ICU LOS vs. the lower 75th percentile from both the ANN training model and the cross-validated model. The threshold for demarcation of the upper quartile for observed ICU LOS for our data is 74.6 hours. Statistically strong odds ratios were found for predicting patients that would be in the upper quartile of ICU LOS for both the training and cross-validated models (Table 2).

**Table 2:** Odds Ratios for Prediction that a Patient would have ICU LOS in the Upper Quartile of Observed Data from both the Trained and Cross-Validated ANN Analyses.

Model	Odds Ratio for LOS Upper Quartile (95% CI)
**Training**	**19.4** (7.5–50.3); **p< 0.0001**
**Cross-Validated “New Patient”**	**9.8** (4.2–22.8); **p< 0.0001**

**Fig 8:**
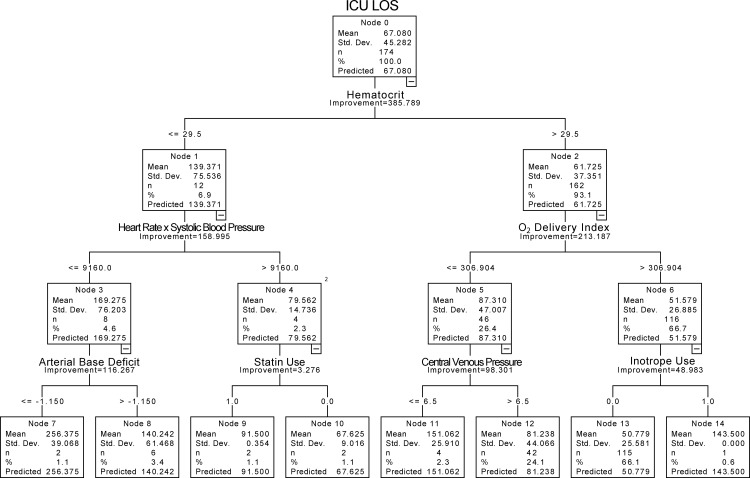
The Final Decision Tree Model that was Assessed for Prediction of ICU LOS.

**Fig 9:**
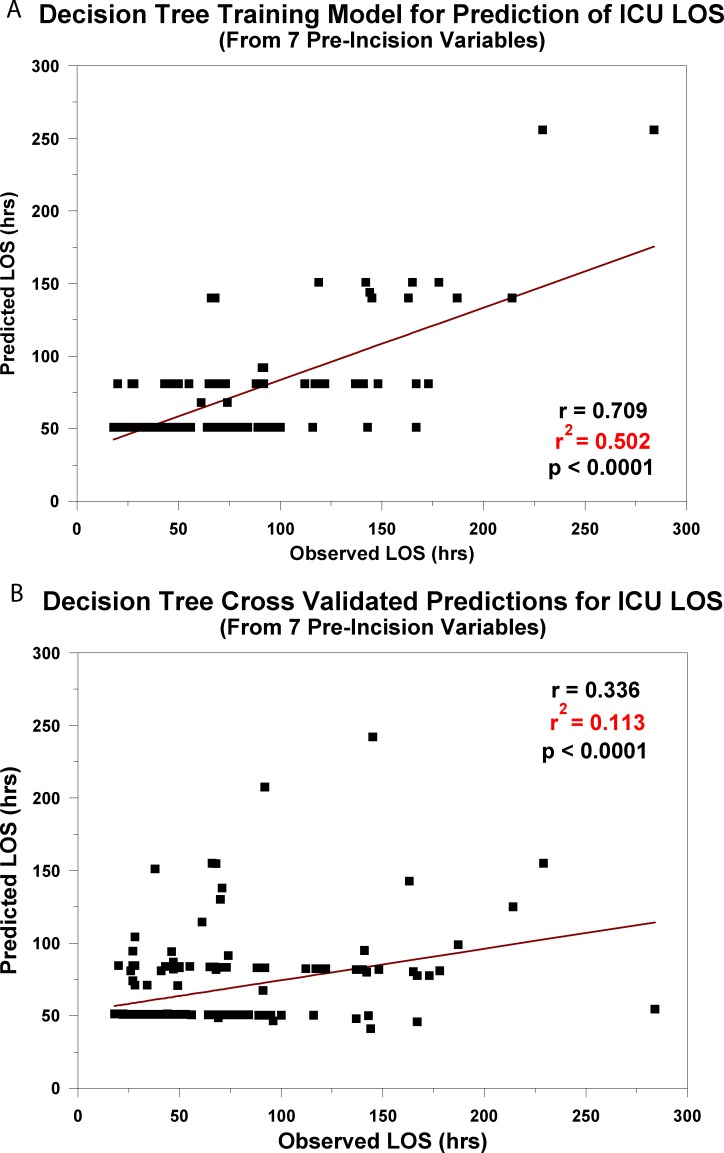
A. Decision Tree Training Model for Prediction of ICU LOS. The regression relationship between the predicted ICU length of stay from 7 pre-incision factors and the observed ICU length of stay from the training model generated by the SPSS Decision Tree module. B. Cross-Validated Predictions for ICU LOS. The cross-validated result of the training model when applied to new ("untrained") patient data.

We also carried out secondary analyses of our data with the SPSS “Decision Tree” algorithm and with “Random Forest” modeled in R. The decision tree analysis was initiated with the original 36 input variables and was assessed for strength of the prediction accuracy by cross-validation of successive levels of branching. We found that four levels of branching produced the greatest prediction accuracy. This tree is presented in Fig 8. It utilized 7 of the input variables at this optimal training level. It shared only 3 of the same variables that were modeled with ALM and ANN: hematocrit (HCT); oxygen delivery index; use of inotropic agents. The prediction accuracy for decision tree in the training algorithm, r^2^ = 0.502, p <0.0001, (Fig 9A) was similar to that for ANN; however, cross-validation with the same “leave-three-out” procedure as described above produced a considerably weaker prediction accuracy (r^2^ = 0.113, p <0.0001; Fig 9B) than that found with ANN.

**Fig 10:**
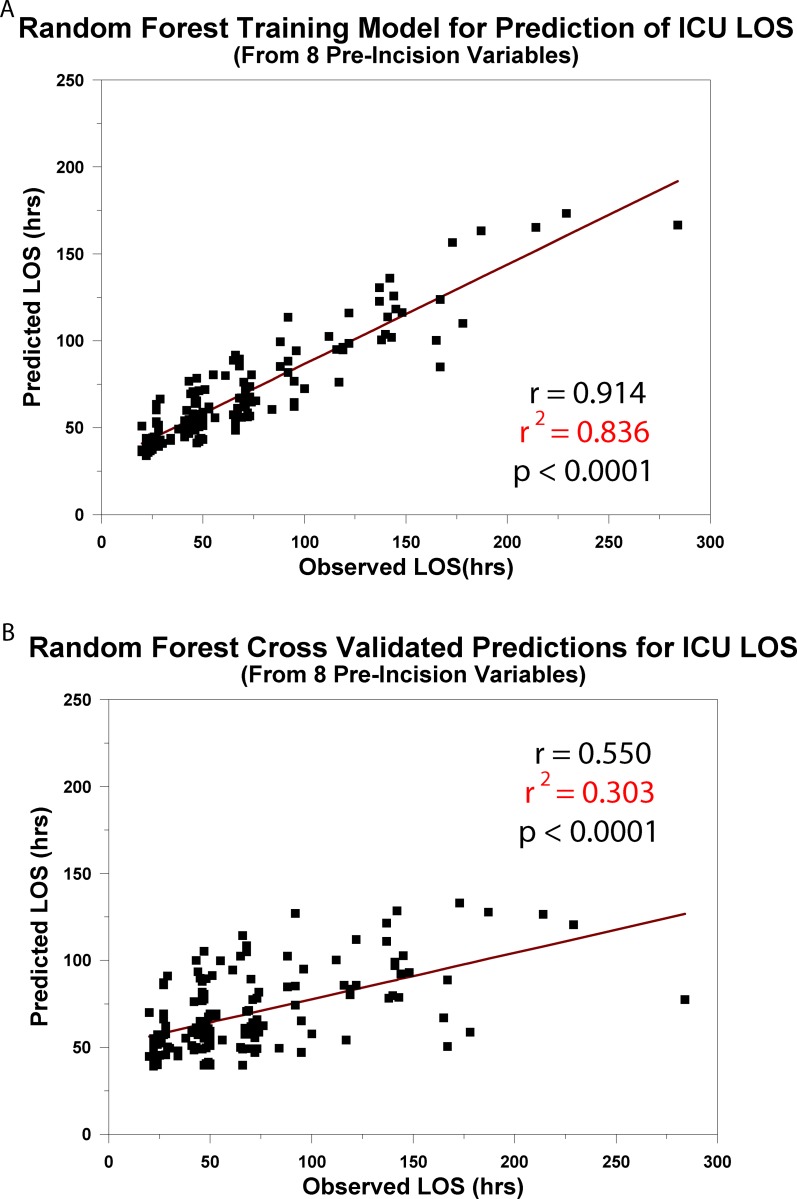
A. Random Forest Training Model for Prediction of ICU LOS. The regression relationship between the predicted ICU length of stay from 8 pre-incision factors and the observed ICU length of stay from the training model. B. Cross-Validated Predictions for ICU LOS. The cross-validated result of the training model when applied to new ("untrained") patient data.

Finally, for the Random Forest modeling, we started with the 8 input variables modeled with ALM and ANN. We used default values for the Random Forest modeling function [12] running in the R software environment [16], yielding a Random Forest model consisting of 500 decision trees. The Random Forest model fit the data with r^2^ = 0.836, p <0.0001 (Fig 10A). The prediction accuracy, evaluated using leave-one-out cross-validation was weaker (r^2^ = 0.303, p <0.0001; Fig 10B) than that found with ANN.

**A** comparison of the prediction accuracies that were found with the four models that were studied is presented in Table 3.

**Table 3:** Comparison of the Accuracies for the Four Models used to Predict ICU LOS.

Model	Training r^2^	Cross Validation r^2^
**Automatic Linear Modeling**	**0.356**	**0.200**
**Artificial Neural Network**	**0.535**	**0.410**
**Decision Tree**	**0.502**	**0.113**
**Random Forest**	**0.836**	**0.303**

All r^2^ values p < 0.0001

## Discussion and Conclusions

We have attempted to develop a neural network to model a continuous measure of hours of ICU LOS following cardiac surgery. It must be noted that non-neural network models have been successfully applied to prediction of ICU-LOS following cardiac surgery. A large prospective study by Barili et al. [17], utilizing both pre-operative variables and surgical data, identified four factors by regression modeling that significantly increased the risk of a prolonged ICU stay following cardiac surgery: Critical preoperative state; surgical emergency; poor left-ventricular function; and elevated serum creatinine. A critical/emergency preoperative state (represented by the need for an intra-aortic balloon pump and preoperative use of positive inotropic agents in our results) as well as elevated serum creatinine were also identified in our analyses. Najafi and Goodarzynejad [18] analyzed factors that would predict ICU-LOS of greater or less than 48 hours. They applied multivariate analysis to pre-operative, intra-operative, and post-operative factors, extending to 6 hours post-operatively for arterial gas analyses and 24 hours for insulin intake. Six factors were independent predictors of ICU-LOS greater than 48 hours: Intra-aortic balloon pump; New York Heart Association functional class; post-operative arrhythmia; 24-hour average insulin intake; mean 6-hour base excess; and a surgeon category.

In contrast to some of the neural network studies referenced above [9–11], we focused on the earliest factors that could be modeled for prediction accuracy. Thus, we limited the variables to those that could be obtained pre-incision. Efforts to identify a set of factors that might be expected intuitively to be useful as predictors of our outcome measure proved difficult. Instead, we subjected our data set of 36 variables (Fig 1) to analysis by the Automatic Linear Modeling module of the IBM-SPSS 21 software. Eight factors were identified that had statistically significant (or approaching significant) associations with ICU LOS (Table 1). It was found that the t statistic and the fractional importance in the ALM model were closely correlated (Fig 2). (The application of the weighted coefficients to determination of ICU LOS is presented in S1 Appendix.)

The trained ALM model for prediction of ICU LOS is presented in Fig 3A (r^2^ = 0.356, p <0.0001). As discussed above, we used a cross-validation approach to test the accuracy of predictions on “untrained” patient data. In this case, repeated sets of data from 3 patients were tested with the trained model obtained from all but those 3 patients. The accuracy of the predictions on “untrained” data was r^2^ = 0.200, p <0.0001 (Fig 3B).

The same 8 factors identified by linear modeling were used in the neural network modeling. It is possible that the inclusion of additional variables might produce even stronger predictive neural network models than we obtained, however, we felt that a direct comparison with the linear modeling would be of interest at this stage of our evaluation of the two models. The neural network trained the data set with an r^2^ value of 0.535, p <0.0001 (Fig 5A). Cross-validation of an ensemble of trained models on repeated sets of 3 untrained patient data, as noted above, produced a predicted r^2^ value of 0.410, p <0.0001 (Fig 5B). The more than twice stronger prediction accuracy of the neural network over the linear model (r^2^ 0.410 vs 0.200) would appear to be related to the contribution of the interactions among the variables to the final predictions. In addition, the rank order of the relative importance of the 8 pre-incision variables to ICU LOS was not identical (Table 4). Presumably, this is again related to the neural network’s capability to capture nonlinear effects, including higher order interactions; linear modeling only considers the strengths of the correlations of the individual factors with the target outcome, ICU LOS.

**Table 4:** Fractional Importance differences between ALM and ANN.

ALM Factor	Fractional Importance %	ANN Factor	Fractional Importance %
**IABP**	**24.2**	**Age**	**24.0**
**O**_**2**_ **Delivery Index**	**27.8**	**O**_**2**_ **Delivery Index**	**21.2**
**Age**	**16.1**	**HCT**	**14.7**
**Pre-incision Ionotropes**	**13.8**	**IABP**	**14.2**
**HCT**	**11.4**	**Arterial pCO**_**2**_	**7.4**
**Creatinine ≥ 1.3**	**6.4**	**Pre-incision Ionotropes**	**7.3**
**Gender**	**5.4**	**Creatinine ≥ 1.3**	**6.4**
**Arterial pCO**_**2**_	**4.9**	**Gender**	**4.8**

Analysis of the 36 input variables with the use of Decision Tree or Random Forest demonstrated that these models could produce training algorithms that had approximately the same or greater predictive accuracy as ANN (Figs 9A and 10A); however, they cross-validated with less accuracy (Figs 9B and 10B; Table 3). It is not suggested that Decision Tree or Random Forest are weaker predictive analytical tools; it is known that performance may vary with the characteristics of the database [19].

It should be emphasized that the predictions of ICU LOS from the neural network models are not calculated from the fractional importance presented in Fig 7 or Table 4. The hours of ICU LOS for the ANN modeling is based on parameter weights (e.g., as in Fig 6) and the algorithm presented in S2 Appendix. The fractional importance measures of Fig 7 or Table 4, also termed “sensitivity” measures, (S3 Appendix) may be useful in identifying factors that warrant clinical consideration in terms of strengths or risks.

It would be expected that a larger data base would yield still stronger prediction accuracy. As new patients are added to the data base, the ICU LOS for each new patient can be predicted from ensembles of trained models from our entire data set. Also, each new patient will next become part of the training set. In principle, each new patient should add to the training experiences such that it becomes increasingly more likely that data from a new untrained patient will already have a close counterpart in the trained data base. At a clinical practice level, for example, data for the 8 input variables can be entered in the software (manually or from an electronic record) and the predicted ICU LOS can be scored from the average output of an ensemble of models generated from the entire dataset. As noted above, we include 10% testing in training the models. This represents our “final” ANN model at this point. The inherent speed of the calculations would provide an almost immediate prediction.

In regard to the possible potential value of neural networks to anticipate high risk patients, our cross-validated network stratified the patients in the upper quartile of observed ICU LOS with an odds ratio of 9.8, p <0.0001 (Table 2).

In conclusion, we believe that our experience with neural network modeling with a relatively small data base demonstrates the potential strength of this approach for identifying and weighting prognosticators of outcomes. The results demonstrate that 41% (the r^2^ value from the neural network cross-validation) of the variation in ICU LOS among these cardiac surgical patients was influenced by 8 pre-incision factors. In one sense, this is quite substantial considering the possible contributions from the surgery itself, the surgical team personnel (surgeons, anesthesiologists, perfusionists, nurses), and the ICU environment and staff. In terms of our outcome measure of ICU LOS, it may have value in identifying the risk of postoperative morbidity. Such analyses may also have economic implications in relation to expected costs and utilization of ICU facilities.

## Limitations

The most serious limitation of this study is the relatively small data base that could be collected during the period that we had IRB approval in place and patient files that had not yet been designated for off-campus storage. However, as noted above, the results give encouragement that modeling of even modest size data bases, which are common in pilot studies, can give indications of possibly worthwhile further investigation. It is also obvious that these studies did not approach the development of new paradigms of predictive modeling, such as those of Zhang et al. [20] and Deng et al. [21]. The paper by Zhang and coworkers presented the evidence that their advanced neural network model, “Extreme Machine Learning,” generally outperformed the more conventional neural network model represented in this present study in classification of several cancer diagnoses from microarray gene expression data [20]. A most recent study by Deng et al. employs still more original algorithm development to classify positive and negative diagnoses in epilepsy, breast cancer and heart disease [21]. Our work is limited to the application of open source software in identifying possibly important associations between patient characteristics and clinical outcomes; however, such studies are often useful in identifying risk factors, guiding treatments, and focusing further investigations.

## Supporting Information

S1 Appendix Application of the coefficients generated in the Automatic Linear Modeling module to calculate the predicted ICU length of stay. (TIF)

S2 Appendix Algorithm for application of the parameter weights generated in the Artificial Neural Network modeling module to calculate the predicted ICU length of stay. (TIF)

S3 Appendix Algorithm for calculation of the relative importance of the input variable (Predictor Importance) in Artificial Neural Network modeling. (TIF)

S4 Appendix Complete database for these studies. (CSV)
