# Supplementary figures and images for: Neural Network Prediction of ICU Length of Stay Following Cardiac Surgery Based on Pre-Incision Variables
Source: PLoS One. 2015 Dec 28;10(12):e0145395. doi: 10.1371/journal.pone.0145395 (PMC4692524; doi:10.1371/journal.pone.0145395)

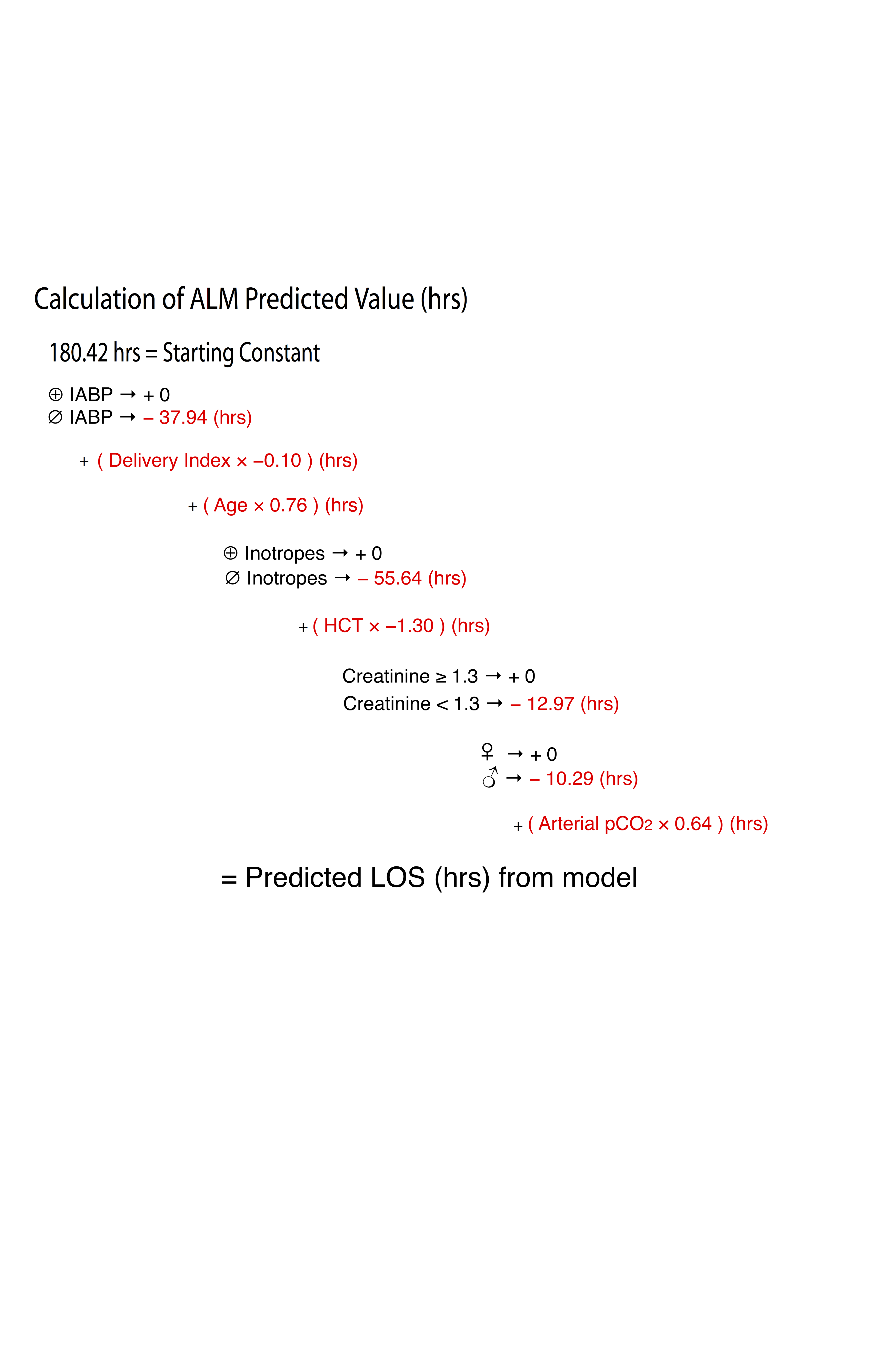

Supplement: S1 Appendix — (TIF) [file pone.0145395.s001.tif]

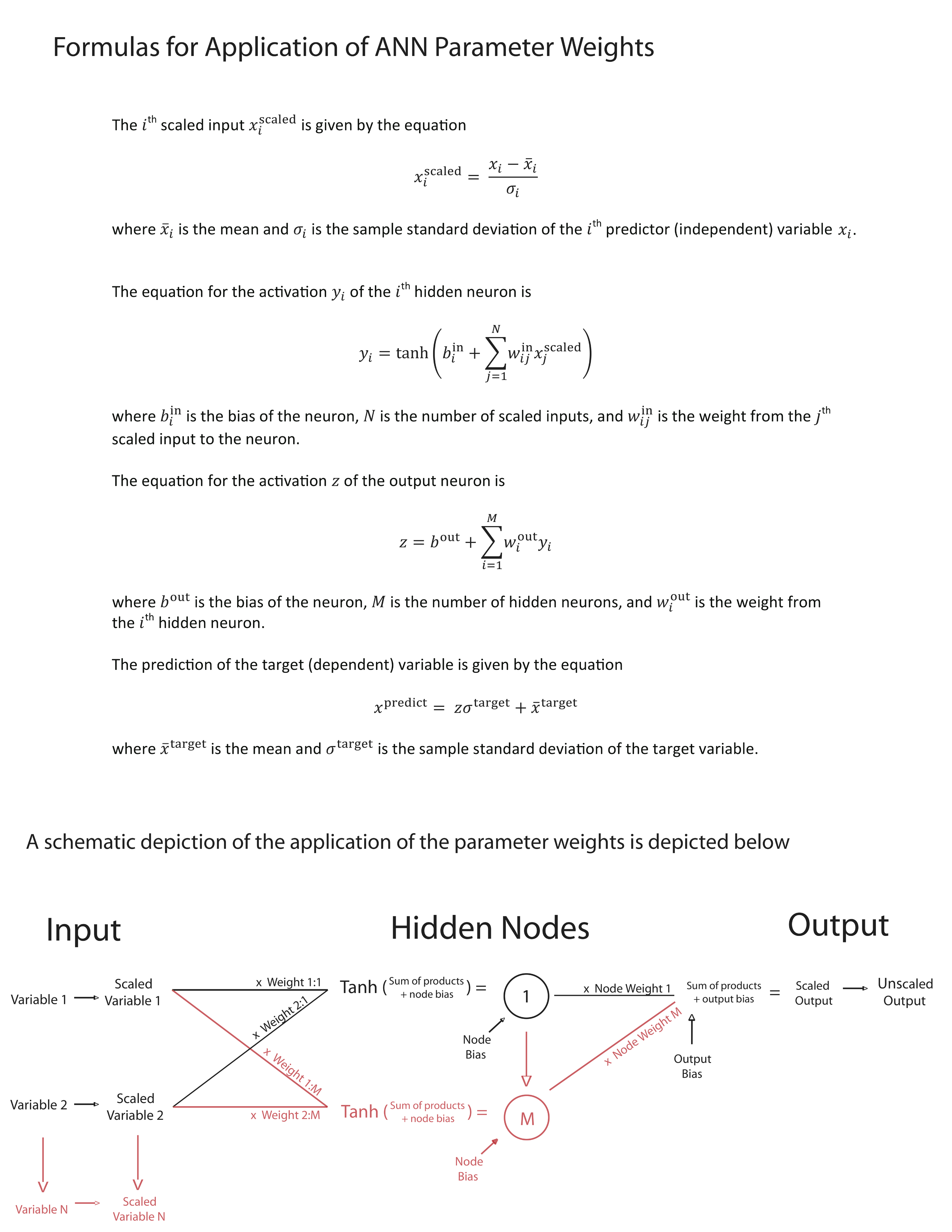

Supplement: S2 Appendix — (TIF) [file pone.0145395.s002.tif]

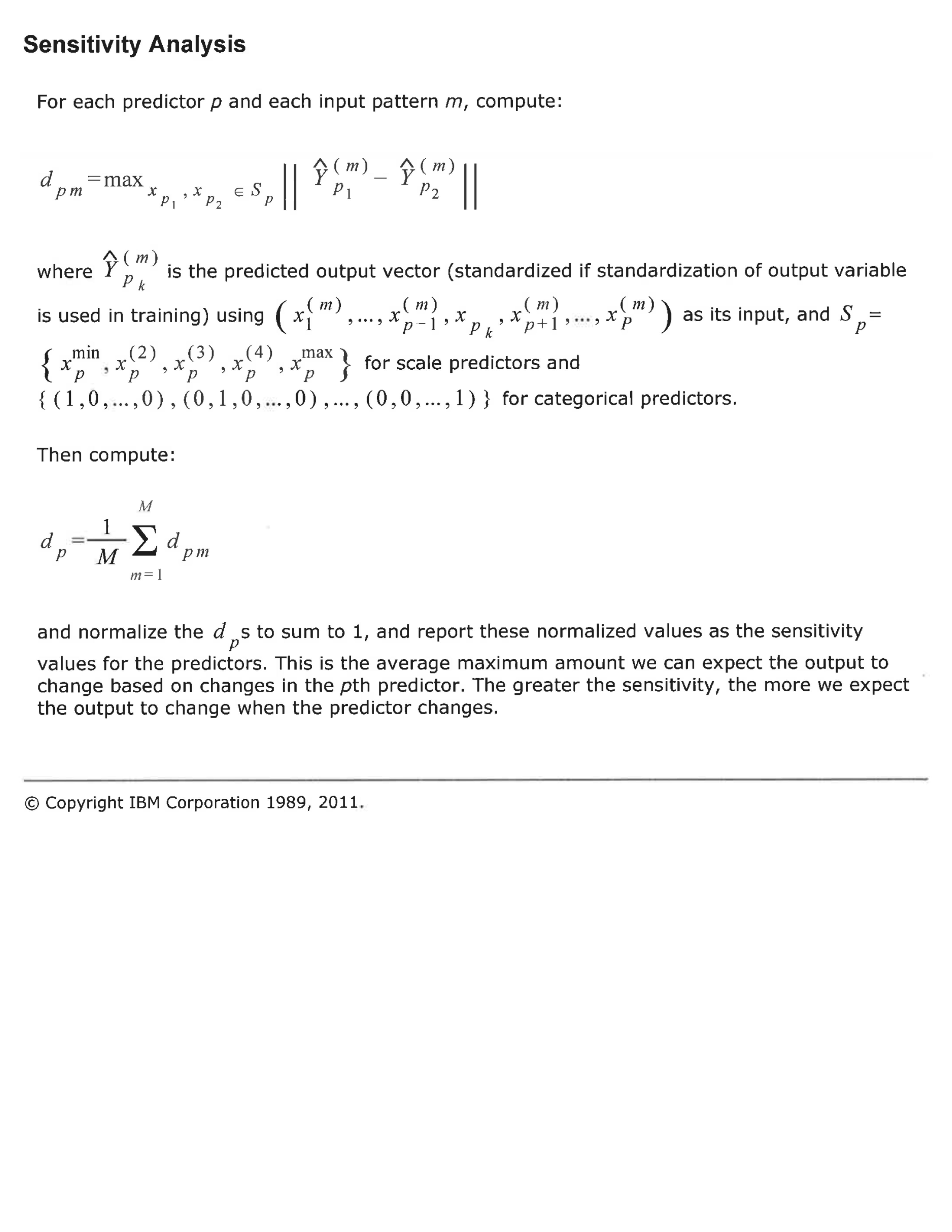

Supplement: S3 Appendix — (TIF) [file pone.0145395.s003.tif]
